# Safety and Operative Burden of Immediate Lymphatic Reconstruction During Breast Cancer Surgery With Concurrent Breast Reconstruction: A 13‐Year ACS‐NSQIP Analysis

**DOI:** 10.1002/micr.70327

**Published:** 2026-11-08

**Authors:** Georgios Karamitros, Georgios Bouloukakis, Galen Perdikis, Yvonne M. Rasko, George Kokosis, Roman Skoracki, Gregory A. Lamaris

**Affiliations:** ^1^ Department of Plastic Surgery Vanderbilt University Medical Center Nashville Tennessee USA; ^2^ Department of Surgery, College of Medicine State University of New York, Downstate Health Sciences University Brooklyn New York USA; ^3^ Division of Plastic and Reconstructive Surgery University of Maryland Medical Center Baltimore Maryland USA; ^4^ Division of Plastic and Reconstructive Surgery Rush University Medical Center Chicago Illinois USA; ^5^ Department of Plastic Surgery The Ohio State University Columbus Ohio USA; ^6^ Division of Plastic and Reconstructive Surgery R. Adams Cowley Shock Trauma Center, University of Maryland Medical Center Baltimore Maryland USA

**Keywords:** breast reconstruction, immediate reconstruction, lymphatic reconstruction, mastectomy, NSQIP, reconstructive surgery

## Abstract

**Background:**

Immediate lymphatic reconstruction (ILR) is increasingly performed during axillary lymph node dissection to reduce breast cancer‐related lymphedema. Prior national studies evaluated broader ALND populations, while the perioperative safety and operative‐duration profile of ILR specifically during concurrent breast reconstruction remains incompletely defined. We evaluated 30‐day safety and total operative duration associated with ILR in women undergoing breast cancer surgery with concurrent reconstruction.

**Methods:**

We performed a retrospective cohort study using ACS‐NSQIP data from 2012 to 2024. Adult women with breast malignancy undergoing breast extirpative surgery with same‐encounter implant‐based or autologous reconstruction were included. ILR was identified using a published CPT‐based algorithm. The primary outcome was any 30‐day postoperative complication. Secondary outcomes included wound complications, surgical‐site infection (SSI), return to the operating room, readmission, operative time, and length of stay. Temporal efficiency was assessed using an ILR‐by‐year interaction.

**Results:**

Among 65,437 women, 367 (0.56%) underwent ILR. Use increased from 0.03% in 2012 to 1.57% in 2024. Documented ALND was present in 72.2% of ILR recipients and 18.7% of nonrecipients. ILR was not associated with any 30‐day complication (adjusted odds ratio [aOR] = 0.98; 95% CI = 0.71–1.35; *p* = 0.898), wound complications (aOR = 1.11; 95% CI = 0.75–1.65; *p* = 0.597), SSI (aOR = 0.88; 95% CI = 0.56–1.40; *p* = 0.600), return to the operating room (aOR = 0.92; 95% CI = 0.62–1.37; *p* = 0.681), or readmission (aOR = 1.05; 95% CI = 0.65–1.70; *p* = 0.834). ILR was associated with a 90.4‐min longer total operative duration (95% CI = 80.1–100.6; *p <* 0.001), without a difference in postoperative length of stay (adjusted mean difference, 0.03 days; 95% CI = −0.21 to 0.26; *p* = 0.832). The operative‐duration difference did not attenuate over time (interaction = 0.44 min/year; 95% CI = −2.89 to 3.78; *p* = 0.796).

**Conclusions:**

ILR was not associated with increased adjusted 30‐day morbidity but was associated with longer total operative duration. These findings support short‐term perioperative safety in patients undergoing concurrent reconstruction but do not establish long‐term effectiveness in preventing breast cancer‐related lymphedema.

## 1 Introduction

Breast cancer‐related lymphedema remains one of the most consequential survivorship complications after axillary treatment. Although axillary de‐escalation has reduced exposure to axillary lymph node dissection (ALND), many patients who require ALND also receive systemic therapy and/or regional nodal irradiation, resulting in cumulative treatment‐related risk for breast cancer‐related lymphedema (Rockson 2018; Johnson et al. 2019; Wang et al. 2025). For the practicing breast and reconstructive surgeon, the clinical question is no longer whether lymphedema matters, but whether an added preventive microsurgical procedure can be incorporated safely into an already complex oncologic‐reconstructive operation. Immediate lymphatic reconstruction (ILR), also termed the lymphatic microsurgical preventive healing approach (LYMPHA), addresses this problem at the index operation by anastomosing transected upper‐extremity lymphatics to adjacent venules during ALND (Johnson et al. 2019; Boccardo et al. 2009, 2011). Early institutional studies, meta‐analytic data, and recent national claims analyses suggest that ILR may reduce subsequent lymphedema and that its use has increased rapidly in contemporary practice (Johnson et al. 2019; Shah et al. 2026; Cho et al. 2025). Yet prevention benefit alone is insufficient to justify broad adoption. Because ILR is performed prophylactically, surgeons must also know whether it increases short‐term wound morbidity, reoperation, readmission, length of stay, or operative burden.

This safety question remains unresolved. Most available ILR studies are single‐institution series or claims‐based analyses designed primarily to evaluate lymphedema incidence rather than perioperative surgical morbidity. As a result, the evidence available for operative counseling is incomplete: the same patient who may benefit from lymphedema prevention must also be counseled about surgical‐site infection (SSI), dehiscence, return to the operating room, readmission, and additional anesthesia time. These outcomes are immediate, measurable, and directly relevant to surgical decision‐making, resource allocation, and payer evaluation.

We therefore used the American College of Surgeons National Surgical Quality Improvement Program (ACS‐NSQIP) dataset from 2012 to 2024 to evaluate the national perioperative safety profile of ILR among women undergoing breast extirpative surgery with immediate reconstruction. By focusing on short‐term outcomes rather than lymphedema efficacy, this study provides complementary evidence for surgeons deciding whether ILR can be safely integrated into routine breast cancer reconstruction pathways.

## 2 Methods

### 2.1 Study Design and Data Source

We performed a retrospective cohort study using ACS‐NSQIP Participant Use Files from 2012 to 2024. ACS‐NSQIP is a clinically audited registry of standardized perioperative variables and 30‐day outcomes across participating hospitals (Karamitros, Catic, Grant, et al. 2026; Raval and Pawlik 2018; Awad et al. 2015; Dindo et al. 2004; Karamitros, Catic, Gergoudis, et al. 2026). The study used de‐identified data and received exempt review from the Vanderbilt University Medical Center Institutional Review Board (#260824). Reporting followed STROBE, RECORD, TRESS, and CLARITY‐SURG principles (Von Elm et al. 2007; Benchimol et al. 2015; Karamitros, Catic, Goulas, et al. 2026; Karamitros, Johnson, Gergoudis, et al. 2026).

### 2.2 Study Population

Eligible patients were women aged 18 years or older with an ICD‐coded breast malignancy (ICD‐10 C50.x; ICD‐9 174.x before October 2015) who underwent breast extirpative surgery (CPT 19301–19307) with same‐encounter implant‐based or autologous reconstruction (CPT 19340, 19342, 19357, 19361, 19364, and 19367–19369). Patients were excluded for male sex, missing core demographic fields required for adjustment, or nonelective operative indication. The final cohort included 65,437 patients (Figure 1).

**FIGURE 1:**
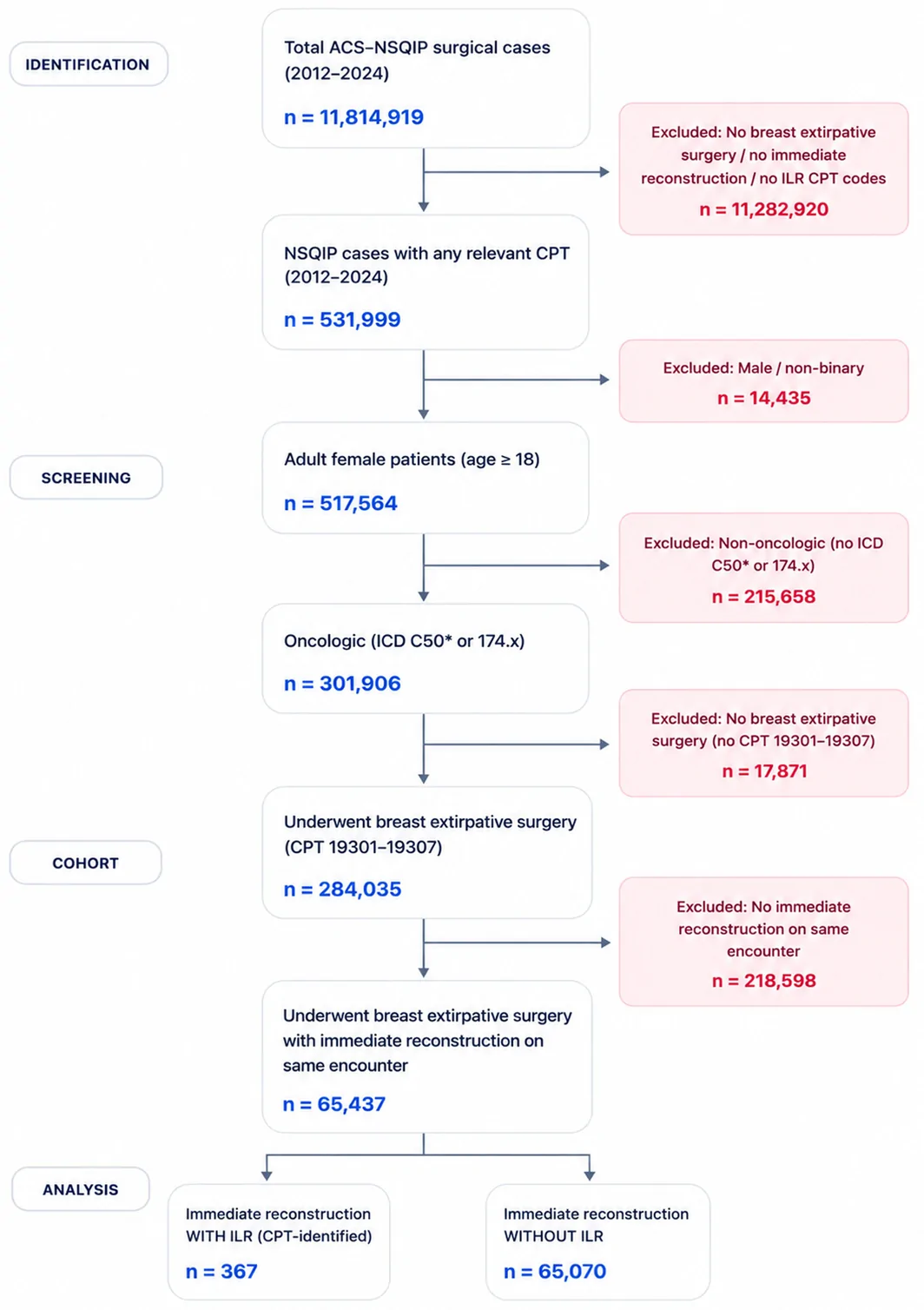
Study cohort selection and classification. *Note:* Flow diagram demonstrating cohort derivation from the American College of Surgeons National Surgical Quality Improvement Program (ACS‐NSQIP) database (2012–2024). Starting from 11,814,919 surgical cases, eligible patients were restricted to female adult patients with oncologic breast diagnosis codes, breast extirpative surgery (CPT 19301–19307), and immediate breast reconstruction on the same operative encounter. The final cohort included 65,437 patients: 367 with immediate lymphatic reconstruction (ILR) and 65,070 without ILR. The asterisk (*) denotes a wildcard; C50 includes all ICD‐10‐CM diagnosis codes beginning with C50 (C50.x).

### 2.3 Exposure

The exposure was ILR, also termed LYMPHA, performed during the same encounter as breast extirpative surgery and immediate reconstruction. ILR was identified by the presence of at least one prespecified CPT code in any procedure field: 38999, 35201, 35206, 38308, 35236, 38305, or 36833. Because CPT 38999 is nonspecific, sensitivity analyses excluded CPT 38999 and, separately, the infrequently used vascular codes CPT 35201 and 38305. The complete coding audit is provided in Table 1.

**TABLE 1:** Baseline characteristics by immediate lymphatic reconstruction status.

Characteristic	Overall	No ILR	ILR	*p*
No. of patients	65,437	65,070	367	
Age, years, median [IQR]	51 [44, 60]	51 [44, 60]	49 [40, 58]	< 0.001
Age category				< 0.001
18–39	9145 (14.0%)	9055 (13.9%)	90 (24.5%)	
40–49	20,227 (30.9%)	20,133 (30.9%)	94 (25.6%)	
50–59	19,392 (29.6%)	19,283 (29.6%)	109 (29.7%)	
60–69	12,825 (19.6%)	12,765 (19.6%)	60 (16.3%)	
70+	3848 (5.9%)	3834 (5.9%)	14 (3.8%)	
Race/ethnicity				0.042
Asian	3220 (4.9%)	3207 (4.9%)	13 (3.5%)	
Black	6504 (9.9%)	6451 (9.9%)	53 (14.4%)	
Hispanic	5390 (8.2%)	5354 (8.2%)	36 (9.8%)	
Other	557 (0.9%)	555 (0.9%)	2 (0.5%)	
Unknown	7079 (10.8%)	7048 (10.8%)	31 (8.4%)	
White	42,687 (65.2%)	42,455 (65.2%)	232 (63.2%)	
BMI (kg/m^2^); median [IQR]	27 [23, 32]	27 [23, 32]	28 [25, 33]	0.001
BMI category				0.015
Missing	273 (0.4%)	269 (0.4%)	4 (1.1%)	
Normal	22,611 (34.6%)	22,510 (34.6%)	101 (27.5%)	
Obese	21,236 (32.5%)	21,100 (32.4%)	136 (37.1%)	
Overweight	20,466 (31.3%)	20,345 (31.3%)	121 (33.0%)	
Underweight	851 (1.3%)	846 (1.3%)	5 (1.4%)	
Current smoker, *n* (%)	6046 (9.2%)	6019 (9.3%)	27 (7.4%)	0.247
Diabetes, *n* (%)	4277 (6.5%)	4258 (6.5%)	19 (5.2%)	0.342
Hypertension, *n* (%)	15,981 (24.4%)	15,893 (24.4%)	88 (24.0%)	0.891
COPD, *n* (%)	468 (0.7%)	467 (0.7%)	1 (0.3%)	0.530
Congestive heart failure, *n* (%)	117 (0.2%)	117 (0.2%)	0 (0.0%)	1.000
Chronic steroid use, *n* (%)	2040 (3.1%)	2009 (3.1%)	31 (8.4%)	< 0.001
Bleeding disorder, *n* (%)	528 (0.8%)	524 (0.8%)	4 (1.1%)	0.547
Disseminated cancer, *n* (%)	1074 (1.6%)	1063 (1.6%)	11 (3.0%)	0.065
ASA class				< 0.001
1	2969 (4.5%)	2960 (4.5%)	9 (2.5%)	
2	42,521 (65.0%)	42,331 (65.1%)	190 (51.8%)	
3	19,648 (30.0%)	19,482 (29.9%)	166 (45.2%)	
4	247 (0.4%)	246 (0.4%)	1 (0.3%)	
5	2 (0.0%)	1 (0.0%)	1 (0.3%)	
Missing	50 (0.1%)	50 (0.1%)	0 (0.0%)	
Functional status				0.721
Independent	64,953 (99.3%)	64,589 (99.3%)	364 (99.2%)	
Missing	364 (0.6%)	361 (0.6%)	3 (0.8%)	
Partially dependent	115 (0.2%)	115 (0.2%)	0 (0.0%)	
Totally dependent	5 (0.0%)	5 (0.0%)	0 (0.0%)	
Reconstruction modality				0.003
Autologous	8023 (12.3%)	7958 (12.2%)	65 (17.7%)	
Implant	56,085 (85.7%)	55,786 (85.7%)	299 (81.5%)	
Mixed	1329 (2.0%)	1326 (2.0%)	3 (0.8%)	
Breast extirpative procedure				< 0.001
Modified radical	64 (0.1%)	64 (0.1%)	0 (0.0%)	
Modified radical + extended	10,081 (15.4%)	9848 (15.1%)	233 (63.5%)	
Partial	1336 (2.0%)	1334 (2.1%)	2 (0.5%)	
Partial + axillary	347 (0.5%)	342 (0.5%)	5 (1.4%)	
Radical	587 (0.9%)	586 (0.9%)	1 (0.3%)	
Simple	49,835 (76.2%)	49,709 (76.4%)	126 (34.3%)	
Subcutaneous	3187 (4.9%)	3187 (4.9%)	0 (0.0%)	
Axillary surgery				< 0.001
Documented ALND	12,401 (19.0%)	12,136 (18.7%)	265 (72.2%)	
Sentinel/limited, no documented ALND	14,071 (21.5%)	14,034 (21.6%)	37 (10.1%)	
Other axillary, no documented ALND	23,143 (35.4%)	23,100 (35.5%)	43 (11.7%)	
No documented axillary surgery	15,822 (24.2%)	15,800 (24.3%)	22 (6.0%)	
Operative time, min, median [IQR]	212 [158, 287]	212 [158, 286]	318 [253, 429]	< 0.001
Operative year				< 0.001
2012	3885 (5.9%)	3884 (6.0%)	1 (0.3%)	
2013	4697 (7.2%)	4689 (7.2%)	8 (2.2%)	
2014	4715 (7.2%)	4706 (7.2%)	9 (2.5%)	
2015	5166 (7.9%)	5150 (7.9%)	16 (4.4%)	
2016	5423 (8.3%)	5406 (8.3%)	17 (4.6%)	
2017	5675 (8.7%)	5663 (8.7%)	12 (3.3%)	
2018	5403 (8.3%)	5387 (8.3%)	16 (4.4%)	
2019	5431 (8.3%)	5392 (8.3%)	39 (10.6%)	
2020	4996 (7.6%)	4964 (7.6%)	32 (8.7%)	
2021	5252 (8.0%)	5210 (8.0%)	42 (11.4%)	
2022	5141 (7.9%)	5089 (7.8%)	52 (14.2%)	
2023	4763 (7.3%)	4717 (7.2%)	46 (12.5%)	
2024	4890 (7.5%)	4813 (7.4%)	77 (21.0%)	

*Note:* Continuous variables are reported as median [interquartile range] and categorical variables as *n* (%). The four‐level axillary classification is presented descriptively. Adjusted models used the binary documented‐ALND variable.

Abbreviations: ALND, axillary lymph node dissection; ASA, American Society of Anesthesiologists; BMI, body mass index; COPD, chronic obstructive pulmonary disease; ILR, immediate lymphatic reconstruction; IQR, interquartile range.

### 2.4 Outcomes

The primary outcome was any 30‐day postoperative complication, defined as a composite of superficial incisional SSI, deep incisional SSI, organ/space SSI, wound dehiscence, unplanned return to the operating room, pneumonia, unplanned reintubation, urinary tract infection, systemic sepsis, and septic shock.

Secondary outcomes included wound complications, any‐depth and individual SSI categories, return to the operating room, unplanned 30‐day readmission, operative time, and postoperative length of stay. Mortality was reported descriptively because events were rare. An exploratory panel of individual ACS‐NSQIP complications, including bleeding, venous thromboembolism, and severe systemic events, was used to identify specific safety signals and was considered hypothesis‐generating.

### 2.5 Covariates

The primary adjusted model included age, body mass index, American Society of Anesthesiologists (ASA) physical status, reconstruction modality, documented ALND, and operative year. Reconstruction was categorized as implant‐based, autologous, or mixed.

Documented ALND was defined by CPT 38745, 19302, or 19307 in any procedure field. Encounters were classified as ALND‐positive regardless of concurrent sentinel‐node, lymphatic‐mapping, or other axillary procedures. Because CPT 19307 incorporates modified radical mastectomy with axillary lymphadenectomy, documented ALND represented encounter‐level coding evidence and did not require a separately billed lymphadenectomy code.

For descriptive reporting, axillary surgery was categorized as no documented axillary surgery, sentinel or limited surgery without ALND, other axillary surgery without ALND, or documented ALND. Adjusted full‐cohort models used the binary documented‐ALND variable. CPT 38740, representing superficial axillary lymphadenectomy, was included in the definition of any axillary surgery but excluded from the primary ALND definition; an alternative definition incorporating CPT 38740 was evaluated in sensitivity analysis.

The parsimonious model was selected to limit overfitting while accounting for major factors associated with ILR selection and postoperative morbidity (Aarts 2007). A 15‐covariate model was examined as a sensitivity analysis.

### 2.6 Statistical Analysis

Continuous variables were summarized as median [interquartile range] because their distributions were non‐normal and included right‐skewed measures such as operative duration and length of stay.

Categorical variables were summarized as counts with percentages. Between‐group comparisons used the Wilcoxon rank‐sum test for continuous variables and the chi‐square or Fisher exact test for categorical variables, as appropriate (Divine et al. 2013; Upton 1992).

Multivariable logistic regression was used to estimate associations between ILR and 30‐day binary outcomes, adjusting for the prespecified covariates. Results are reported as adjusted odds ratios (aORs) with 95% confidence intervals. Firth‐penalized logistic regression was used for uncommon outcomes with sparse or zero events in the ILR group (Puhr et al. 2017). Operative time and length of stay were analyzed using multivariable linear regression and reported as adjusted mean differences. Sensitivity analyses restricted the cohort to patients with any documented axillary surgery or documented ALND; limited the study period to 2019–2024; excluded CPT 38999 or CPT 35201 and 38305 from the ILR definition; used the 15‐covariate model; incorporated CPT 38740 into the ALND definition; and applied 1:2 nearest‐neighbor propensity‐score matching (Rassen et al. 2012). Covariate balance after matching was assessed using standardized mean differences, with absolute values below 0.10 indicating acceptable balance.

The clinically focused ALND analysis included encounters with CPT 38745, 19302, or 19307. Because ALND was constant in this cohort, adjusted models included age, body mass index, ASA physical status, reconstruction modality, and operative year.

Temporal operative efficiency was assessed using a multivariable linear model containing ILR status, operative year, and an ILR‐by‐year interaction. Because annual ILR counts were sparse before 2019, Years 2012–2018 were pooled for year‐specific estimates, and 2019–2024 were modeled separately. Operative‐time sensitivity analyses included the 2019–2024 period, era‐based modeling, exclusion of the highest 1% of operative times, log transformation, median regression, restriction to any axillary surgery or documented ALND, stratification by reconstruction modality, and exclusion of CPT 38999.

Primary and prespecified secondary outcomes used two‐sided tests with *p <* 0.05 considered statistically significant. The 15 exploratory complications were evaluated using Bonferroni correction (*α* = 0.0033) and Benjamini–Hochberg false‐discovery‐rate *q* values (Sedgwick 2012; Kwong et al. 2002). Analyses were performed using R version 4.4.

## 3 Results

### 3.1 Cohort and National Adoption

Among 11,814,919 ACS‐NSQIP encounters from 2012 to 2024, 65,437 adult women met eligibility criteria for breast extirpative surgery with immediate reconstruction (Figure 1). Of these, 367 patients (0.56%) underwent CPT‐identified ILR, and 65,070 did not. ILR use increased over the study period, from 0.03% of eligible reconstructions in 2012 to 1.57% in 2024, with the greatest increase observed after 2019 (Figure 2).

**FIGURE 2:**
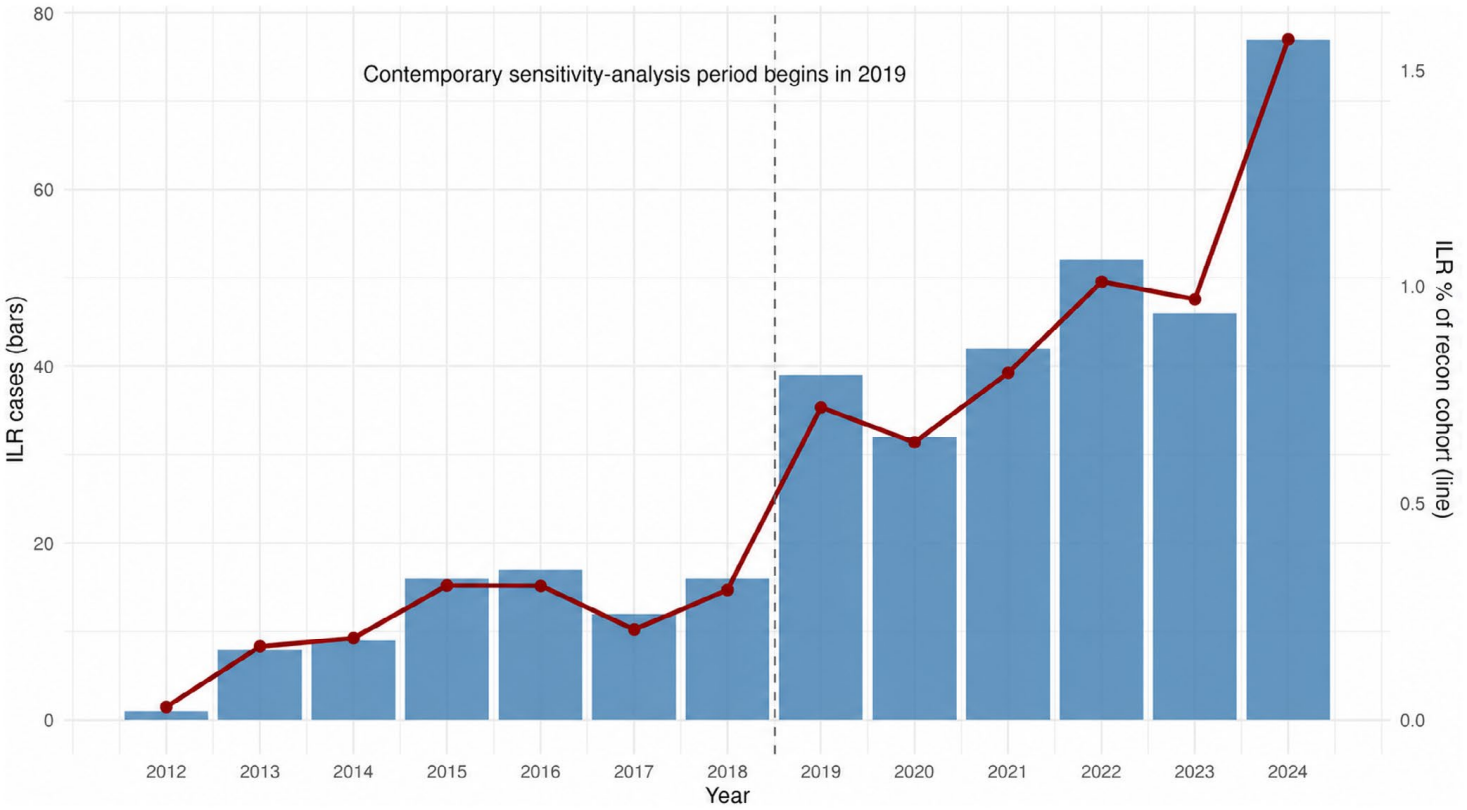
Annual counts of ILR cases, 2012–2024. *Note:* Bars show annual ILR case counts among patients undergoing breast extirpative surgery with concurrent reconstruction; the line shows ILR as a percentage of the annual reconstruction cohort. The dashed vertical line marks the beginning of the contemporary sensitivity‐analysis period (2019–2024). ILR uptake increased from 1 case, 0.03% in 2012 to 77 cases, 1.57% in 2024. ILR indicates immediate lymphatic reconstruction.

### 3.2 Patient and Operative Characteristics

Baseline characteristics suggested selective use of ILR among patients undergoing more extensive axillary surgery (Table 1). Compared with patients who did not undergo ILR, ILR recipients were modestly younger (median age = 49 vs. 51 years; *p <* 0.001), had a slightly higher body mass index (median = 28 vs. 27 kg/m^2^; *p* = 0.001), and had a higher prevalence of chronic steroid use (8.4% vs. 3.1%; *p <* 0.001). ASA physical status also reflected greater comorbidity burden, with ASA Class III recorded in 45.2% of ILR recipients compared with 29.9% of nonrecipients (*p* < 0.001).

The largest between‐group differences were operative and anatomic. Documented ALND was substantially more frequent among ILR recipients than among patients who did not undergo ILR (72.2% vs. 18.7%; *p* < 0.001). Among ILR recipients, 10.1% underwent sentinel or limited axillary surgery without documented ALND, 11.7% underwent another axillary procedure without documented ALND, and 6.0% had no documented axillary procedure. The ILR coding audit, axillary classification, and CPT composition of documented ALND are summarized in Table S1.

ILR was also more commonly performed with autologous reconstruction (17.7% vs. 12.2%). Unadjusted operative time was longer among ILR recipients (median = 318 vs. 212 min; *p* < 0.001). As detailed in the adjusted temporal analysis below, this operative‐time difference persisted at the national registry level throughout the study period, although within‐center improvements in efficiency cannot be excluded.

### 3.3 Adjusted Primary and Secondary Outcomes

In the prespecified adjusted analysis including 65,114 patients, ILR was not associated with the primary composite 30‐day complication outcome (aOR = 0.98; 95% CI = 0.71–1.35; *p* = 0.898) (Table 2). Adjusted estimates for the prespecified secondary clinical outcomes were also not statistically significant, including wound complications (aOR = 1.11; 95% CI = 0.75–1.65; *p* = 0.597), any SSI (aOR = 0.88; 95% CI = 0.56–1.40; *p* = 0.600), return to the operating room (aOR = 0.92; 95% CI = 0.62–1.37; *p* = 0.681), and unplanned 30‐day readmission (aOR = 1.05; 95% CI = 0.65–1.70; *p* = 0.834). Thirty‐day mortality was rare, with 18 deaths overall and no deaths among ILR recipients; an adjusted mortality estimate was therefore not estimable. The principal measurable perioperative difference was operative duration. After adjustment, ILR was associated with a 90.4‐min longer operative time (95% CI = 80.1–100.6 min; *p* < 0.001). Postoperative length of stay did not differ between groups (adjusted mean difference = 0.03 days; 95% CI = −0.21 to 0.26; *p* = 0.832).

**TABLE 2:** Adjusted associations between immediate lymphatic reconstruction and 30‐day outcomes.

Outcome	*N* (events)	Adjusted estimate (95% CI)	*p*
Any 30‐day complication	65,114 (7078)	0.98 (0.71–1.35)	0.898
Wound complication	65,114 (3653)	1.11 (0.75–1.65)	0.597
Any surgical‐site infection	65,114 (3209)	0.88 (0.56–1.40)	0.600
Return to the operating room	65,114 (5079)	0.92 (0.62–1.37)	0.681
Unplanned 30‐day readmission	65,114 (2944)	1.05 (0.65–1.70)	0.834
30‐day mortality	65,114 (18)	Not estimable	—
Operative duration (min)	65,055	+90.37 (+80.10 to +100.64)	< 0.001
Postoperative length of stay (days)	65,085	+0.03 (−0.21 to +0.26)	0.832

*Note:* The exposure contrast is immediate lymphatic reconstruction versus no immediate lymphatic reconstruction. Models were adjusted for age, body mass index, American Society of Anesthesiologists physical status, reconstruction modality, documented axillary lymph node dissection, and operative year. Estimates for binary outcomes are adjusted odds ratios; estimates for operative duration and postoperative length of stay are adjusted mean differences. A total of 30‐day mortality was not estimable because no deaths occurred among ILR recipients.

Abbreviations: ALND, axillary lymph node dissection; ASA, American Society of Anesthesiologists; CI, confidence interval; ILR, immediate lymphatic reconstruction.

### 3.4 Operative Efficiency Over Time

Operative time was modeled using an ILR‐by‐operative‐year interaction to assess whether the adjusted operative‐time difference associated with ILR changed over the study period (Figure 3). In the primary interaction model, ILR was associated with a 90.4‐min longer operative duration (95% CI = 80.1–100.6; *p* < 0.001). The ILR‐by‐year interaction was 0.44 min per year (95% CI = −2.89 to 3.78; *p* = 0.796), providing no evidence that the operative‐time difference decreased as national adoption increased.

**FIGURE 3:**
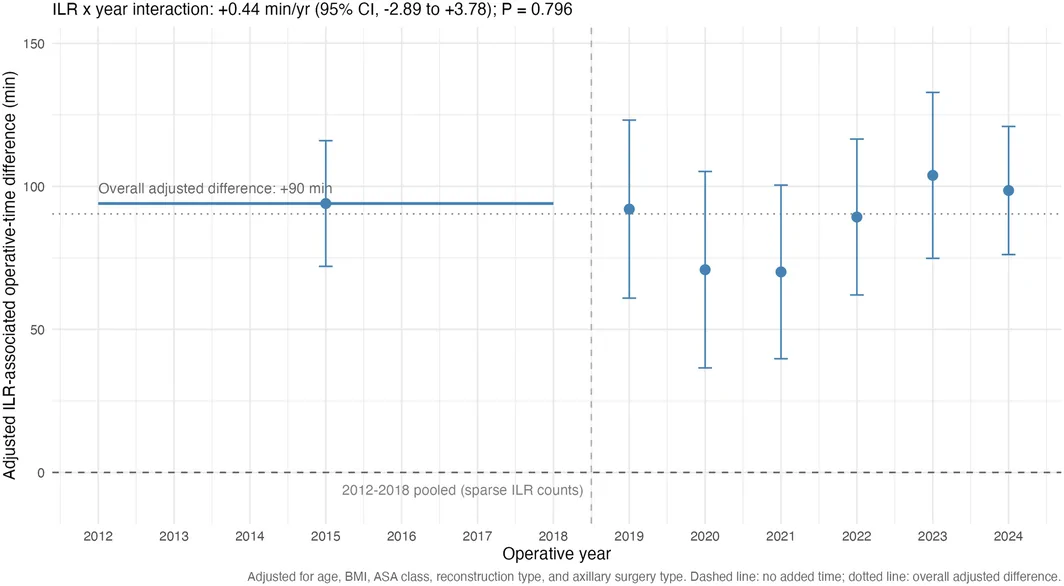
Adjusted difference in total operative duration associated with immediate lymphatic reconstruction over time. *Note:* Points represent the adjusted difference in operative time between patients who did and did not undergo ILR, estimated from multivariable linear regression. Vertical bars represent 95% confidence intervals. Because annual ILR counts were sparse before 2019, the 2012–2018 estimate was pooled; 2019–2024 are shown as single‐year estimates. Models were adjusted for age, body mass index, ASA physical status, reconstruction modality, and documented ALND. The dashed horizontal line marks no added operative time, and the dotted horizontal line marks the overall adjusted mean difference of approximately +90.4 min. The ILR‐associated operative‐time difference remained consistently above zero across the study period, indicating that ILR was associated with longer operations in every modeled period. There was no evidence that this added time decreased as adoption increased: The ILR‐by‐year interaction was +0.44 min per year (95% CI = −2.893 to 3.78; *p* = 0.796). Clinically, this means that the additional operative time required for ILR appeared stable rather than diminishing with national uptake. Thus, the operative‐time burden should be interpreted as a persistent implementation cost of ILR rather than a clearly improving learning‐curve effect.

The operative‐duration finding remained consistent across clinically relevant sensitivity analyses, including restriction to the contemporary 2019–2024 period, exclusion of the highest 1% of operative times, restriction to patients with documented axillary surgery or ALND, stratification by reconstruction modality, and use of the strict ILR definition excluding CPT 38999 (Table S2). Within the documented‐ALND cohort, ILR was associated with a 98.9‐min longer operative duration (95% CI = 86.5–111.4; *p* < 0.001), with no evidence of temporal attenuation (ILR‐by‐year interaction = 1.26 min per year; 95% CI = −2.90 to 5.42; *p* = 0.553) (Table S2).

### 3.5 Robustness and Clinically Relevant Safety Signals

The association between ILR and overall 30‐day morbidity remained consistent across prespecified sensitivity analyses, including alternative ILR coding definitions, contemporary‐era restriction, propensity‐score matching, expanded covariate adjustment, reconstruction modality, and restriction to patients with documented axillary surgery or ALND (Table S2). In the clinically focused documented‐ALND cohort, including 12,320 patients and 262 ILR recipients, ILR was similarly not associated with composite 30‐day morbidity (aOR = 0.87; 95% CI = 0.58–1.32; *p* = 0.518), while total operative duration remained longer (adjusted mean difference = 98.9 min; 95% CI = 86.5–111.4; *p* < 0.001) (Table S3).

Exploratory analyses of individual complications identified wound‐specific findings that warrant cautious interpretation (Table S4; Figure 4). Deep incisional SSI was nominally more frequent after ILR (aOR = 2.43; 95% CI = 1.18–4.98; *p* = 0.016). Wound dehiscence demonstrated a directionally similar but statistically inconclusive association (aOR = 2.00; 95% CI = 0.97–4.11; *p* = 0.059). Neither finding met the prespecified significance threshold after correction for multiple comparisons. Superficial SSI, organ/space infection, bleeding, venous thromboembolism, and severe systemic complications were not significantly increased.

**FIGURE 4:**
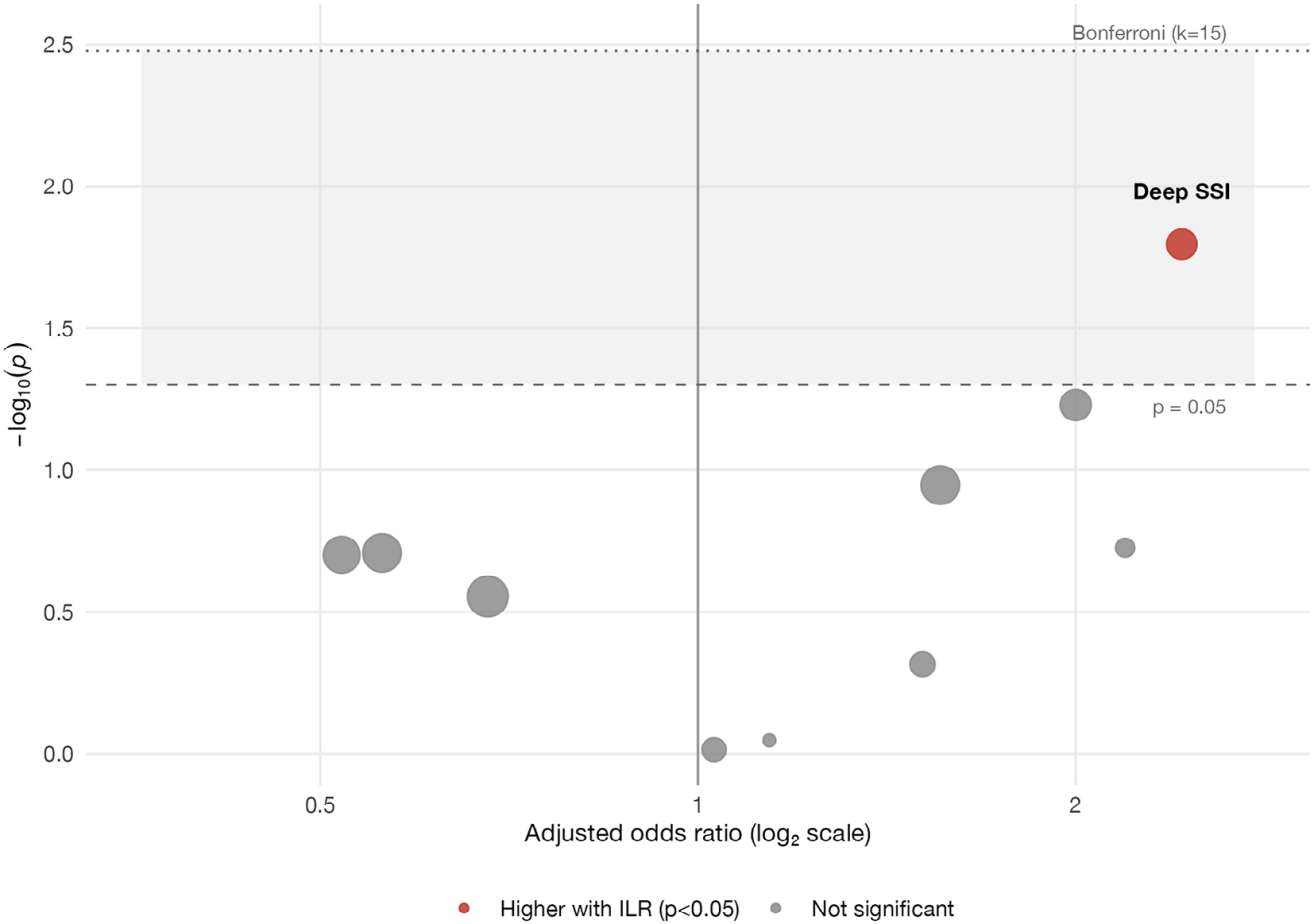
Extended outcomes. *Note:* Volcano plot of exploratory individual 30‐day outcomes after ILR. Each point represents an adjusted odds ratio for a specific complication; the *x*‐axis shows the direction and magnitude of association on a log_2_ scale, and the *y*‐axis shows statistical strength as −log_10_(*p*). Larger points indicate more frequent events. Red points indicate outcomes that were nominally higher with ILR at *p* < 0.05; gray points indicate outcomes that were not statistically significant. The solid vertical line marks no difference between groups, the dashed horizontal line marks *p* = 0.05, and the dotted horizontal line marks the Bonferroni threshold used to account for testing 15 exploratory outcomes. Clinically, this figure shows that most individual complications did not demonstrate a signal for increased risk with ILR. Two wound‐related outcomes—deep incisional surgical site infection, reflecting infection involving deeper soft‐tissue layers rather than only the skin, and wound dehiscence—were numerically higher after ILR and crossed the nominal *p* < 0.05 threshold. However, neither crossed the more stringent multiple‐testing threshold, meaning the findings were not strong enough to be considered reliable evidence of harm after accounting for the number of outcomes examined. These results should therefore be interpreted as hypothesis‐generating wound‐specific safety signals that warrant prospective surveillance, rather than definitive evidence that ILR increases wound complications.

Within the documented‐ALND cohort, deep incisional SSI and wound dehiscence remained directionally elevated but imprecisely estimated; neither association was statistically significant (Table S3).

Given the small number of events and the number of outcomes examined, these wound‐specific findings were considered hypothesis‐generating rather than confirmatory evidence of harm.

## 4 Discussion

In this 13‐year national ACS‐NSQIP cohort of 65,437 women undergoing breast extirpative surgery with immediate reconstruction, CPT‐identified ILR was not associated with increased adjusted 30‐day morbidity, SSI, wound complications, reoperation, readmission, or length of stay. Findings were consistent across alternative coding definitions, propensity‐score matching, contemporary‐era analyses, and restriction to patients with documented ALND. The principal measurable tradeoff was operative time: ILR was associated with approximately 90 additional minutes overall and 99 min among patients with documented ALND, without evidence that this difference decreased as national adoption increased.

Breast cancer‐related lymphedema remains a consequential survivorship complication associated with pain, recurrent infection, functional impairment, psychosocial morbidity, reduced quality of life, and increased health‐care utilization (Rockson 2018; McLaughlin et al. 2008; Ahmed et al. 2008; Beaulac et al. 2002; Shih et al. 2009; Kuruvilla et al. 2022). Although axillary de‐escalation has reduced exposure to ALND, patients who require ALND remain at substantial risk, particularly when regional nodal irradiation is also administered (Johnson et al. 2019; Tsai et al. 2009; Warren et al. 2014). Johnson et al. reported pooled lymphedema incidences of 14.1% after ALND alone and 33.4% after ALND with regional nodal irradiation (Johnson et al. 2019). The relevant implementation question is therefore whether a preventive microsurgical adjunct can be incorporated safely into an already complex oncologic‐reconstructive operation.

ILR was developed to restore lymphatic outflow by anastomosing transected upper‐extremity lymphatics to adjacent venules at the time of ALND (Boccardo et al. 2009, 2011, 2014). Institutional studies and pooled analyses have reported encouraging reductions in subsequent lymphedema, including among patients receiving regional nodal irradiation (Johnson et al. 2019; Feldman et al. 2015; Hahamoff et al. 2019), while national claims data demonstrate increasing adoption and an association with lower odds of postmastectomy lymphedema syndrome (Shah et al. 2026). The present study complements that effectiveness literature by defining the short‐term perioperative safety and resource implications of ILR.

Our findings extend an evolving body of NSQIP evidence on prophylactic lymphatic reconstruction. Chiang et al. first examined prophylactic lymphovenous bypass during ALND using 2013–2019 NSQIP data and reported longer operative duration without increased wound morbidity or reoperation (Chiang et al. 2022). More recently, Pham et al. evaluated a broader 2013–2022 ALND cohort and found that increased unadjusted morbidity was concentrated largely among patients undergoing mastectomy without immediate reconstruction; complication rates were not significantly different in the immediate‐reconstruction subgroup (Pham et al. 2026). The present analysis should therefore be viewed as a reconstruction‐specific extension of prior national ILR studies. By treating concurrent breast reconstruction as the target clinical setting rather than a secondary subgroup, extending observation through 2024, evaluating both implant‐based and autologous reconstruction, and examining a clinically focused cohort with documented ALND, this study defines the perioperative safety and total operative‐duration profile of ILR within the combined oncologic‐reconstructive workflow in which the procedure is increasingly implemented. Collectively, these successive analyses suggest that ILR can be incorporated into contemporary breast cancer reconstruction without a large detectable increase in short‐term morbidity, while remaining associated with longer total operative duration.

The operative‐time difference is particularly relevant because ILR is performed prophylactically, before lymphedema develops. Although the absence of increased reoperation, readmission, SSI, or length of stay is reassuring, an approximately 90‐min longer operation, approaching 99 min in the documented‐ALND cohort, may affect anesthesia exposure, operating‐room scheduling, microsurgical staffing, throughput, institutional cost, and access. The lack of temporal attenuation may reflect a persistent procedural time requirement or the continued entry of new centers early in their implementation experience, offsetting efficiency gains at established programs. Because ACS‐NSQIP lacks longitudinal surgeon‐ and center‐level identifiers, these explanations cannot be distinguished.

The exploratory wound findings warrant caution. Deep incisional SSI was nominally more frequent after ILR, whereas wound dehiscence showed a directionally similar but statistically inconclusive association. Neither finding remained significant after correction for multiple comparisons, and both were imprecise within the documented‐ALND cohort. These signals are clinically plausible given the extent of axillary dissection, lymphatic mapping, microsurgical manipulation, longer operative exposure, and drain dependence, but they do not establish harm. Prospective studies should prespecify axillary wound outcomes, distinguish breast‐site from axillary‐site events, and capture seroma, drain duration, antibiotic practices, and drain‐management protocols.

The findings support an indication‐aligned rather than universal approach to ILR. Documented ALND was present in 72.2% of ILR recipients compared with 18.7% of nonrecipients, consistent with preferential use among patients with greater lymphatic disruption. ALND, regional nodal irradiation, higher body mass index, hypertension, chemotherapy, greater nodal burden, and postoperative complications may further modify absolute lymphedema risk (Rockson 2018; Johnson et al. 2019; Kuruvilla et al. 2022; Tsai et al. 2009; Warren et al. 2014; Kim et al. 2013; Ahmed et al. 2011). Nevertheless, ILR is principally intended as an adjunct once ALND is indicated; its benefit after sentinel lymph node biopsy or targeted axillary dissection remains uncertain. ILR should not influence the oncologic indication for ALND, which must be determined independently according to disease burden and multidisciplinary oncologic management.

National registry and longitudinal institutional studies provide complementary evidence. ACS‐NSQIP can characterize short‐term perioperative safety and operative implementation at scale but cannot determine whether ILR prevents subsequent lymphedema. Accordingly, the present findings should be interpreted as evidence regarding perioperative safety and implementation within concurrent breast reconstruction, not as evidence of long‐term clinical effectiveness.

### 4.1 Limitations

This study has several limitations. First, ILR was identified using CPT codes rather than operative reports or lymphatic mapping data, creating potential exposure misclassification, particularly because CPT code 38999 is nonspecific. Sensitivity analyses excluding CPT 38999 and infrequently used vascular codes produced unchanged results. Moreover, 102 CPT‐identified ILR encounters lacked a prespecified documented‐ALND code, which may reflect incomplete coding, variation in procedures captured by the ILR algorithm, or use outside a conventionally coded complete‐ALND encounter.

Second, ALND ascertainment also relied on encounter‐level CPT coding. Most ALND‐positive ILR encounters were identified through CPT code 19307, which bundles modified radical mastectomy with axillary lymphadenectomy, rather than through a separately billed lymphadenectomy code. Some clinically performed dissections may therefore have been missed, and documented ALND should be interpreted as coding evidence rather than a precise measure of dissection extent. Results were unchanged when CPT code 38740 was incorporated into an alternative ALND definition. Also, ACS‐NSQIP captures total operative duration for the index encounter rather than procedure‐specific start and stop times. Accordingly, the approximately 90‐min difference associated with ILR cannot be attributed entirely to the lymphatic reconstruction itself, particularly because ILR and components of breast reconstruction may be performed concurrently or sequentially within the same operation.

Third, ACS‐NSQIP captures only 30‐day outcomes and does not measure lymphedema, patient‐reported outcomes, quality of life, or cost‐effectiveness. It also lacks granular oncologic, radiation, and technical variables, including nodal burden, ALND indication, systemic therapy, radiation fields, number of nodes removed, lymphatic mapping, bypass configuration, drain duration, seroma, antibiotic protocols, surgeon experience, and use of conventional versus robotic‐assisted microsurgery. Although multivariable adjustment, propensity‐score matching, subgroup analyses, and sensitivity analyses addressed measured differences, residual confounding remains possible (Karamitros et al. 2025). The absence of longitudinal center‐ and surgeon‐level identifiers also prevents separation of within‐center efficiency gains from the entry of new programs early in their learning curves.

Finally, the ILR cohort remained relatively small despite the large overall sample. The study was therefore better powered to exclude large differences in common outcomes than rare complications or modest wound‐specific risks. The deep‐incisional SSI and wound‐dehiscence signals were based on few events, did not remain significant after multiple‐comparison adjustment, and require validation in prospective ILR registries with procedure‐ and site‐specific wound assessment.

## 5 Conclusions

In this national ACS‐NSQIP cohort, ILR was not associated with increased adjusted 30‐day morbidity among patients undergoing breast cancer surgery with concurrent reconstruction, including those with documented ALND. Its principal measurable tradeoff was longer total operative duration, while wound‐specific findings remained hypothesis‐generating. These data support the short‐term perioperative safety of ILR in selected patients while defining the operative‐resource investment associated with its use. Whether this perioperative profile translates into durable lymphedema prevention requires longitudinal evaluation.

## Author Contributions

Concept and design: Georgios Karamitros. Acquisition, analysis, or interpretation of data: Georgios Karamitros and Georgios Bouloukakis. Drafting of the manuscript: Georgios Karamitros, Georgios Bouloukakis. Critical revision of the manuscript for important intellectual content: Georgios Karamitros, Georgios Bouloukakis, Gregory A. Lamaris, Yvonne M. Rasko, George Kokosis, Galen Perdikis, and Roman Skoracki. Supervision: Georgios Karamitros and Gregory A. Lamaris.

## Funding

The authors have nothing to report.

## Ethics Statement

The authors have nothing to report.

## Consent

This study was reviewed and determined to be exempt by the Vanderbilt University Institutional Review Board due to the use of de‐identified data. The American College of Surgeons National Surgical Quality Improvement Program and the hospitals participating in the ACS‐NSQIP are the source of the data used herein; they have not verified and are not responsible for the statistical validity of the data analysis or the conclusions derived by the authors.

## Conflicts of Interest

The authors declare no conflicts of interest.

## Supporting information


**Supplementary Appendix Table S1:** CPT audit for immediate lymphatic reconstruction and documented axillary lymph node dissection.
**Table S2:** Consolidated sensitivity and subgroup analyses of immediate lymphatic reconstruction.
**Table S3:** Outcomes associated with immediate lymphatic reconstruction among patients with documented axillary lymph node dissection.
**Table S4:** Exploratory individual 30‐day outcomes associated with immediate lymphatic reconstruction.

## Data Availability

The data used in this study were obtained from the American College of Surgeons National Surgical Quality Improvement Program (ACS‐NSQIP) Participant Use Files. These data are not publicly available from the authors because access is governed by the American College of Surgeons and requires participation in, or authorized access to, the ACS‐NSQIP program. Researchers interested in accessing these data should apply through the American College of Surgeons in accordance with its data‐use policies.
