# Supplementary material for: Safety and Operative Burden of Immediate Lymphatic Reconstruction During Breast Cancer Surgery With Concurrent Breast Reconstruction: A 13‐Year ACS‐NSQIP Analysis
Source: Microsurgery. 2026 Nov 8;46(8):e70327. doi: 10.1002/micr.70327 (PMC13746070; doi:10.1002/micr.70327)
Supplement: Supplementary file 1 [file MICR-46-e70327-s001.pdf]

6 Supplementary Appendix

The Supplementary Appendix provides supporting analyses focused on the clinical robustness and interpretation of the primary findings. Table S1 summarizes the CPT-based identification of ILR and documented ALND. Table S2 consolidates prespecified subgroup and sensitivity analyses for the primary 30-day morbidity outcome and total operative duration. Table S3 presents exploratory individual 30-day complications, and Table S4 provides detailed outcomes within the clinically focused cohort of patients with documented ALND.

Table S1: CPT Audit for Immediate Lymphatic Reconstruction and Documented Axillary Lymph Node Dissection

Panel A. Prespecified ILR candidate CPT codes

CPT code	CPT rows	Unique cases
38999	185	184
35201	0	0
35206	110	108
38308	127	123
35236	14	13
38305	0	0
36833	2	2

Panel B. ILR candidate code counts by operative year

Year	38999	35201	35206	38308	35236	38305	36833	Any ILR
2012	0	0	0	0	1	0	0	1
2013	0	0	0	8	0	0	0	8
2014	1	0	0	6	2	0	0	9
2015	4	0	1	10	1	0	0	16
2016	2	0	3	12	0	0	0	17
2017	3	0	3	7	0	0	0	12
2018	2	0	11	11	1	0	0	16
2019	22	0	15	16	0	0	0	39
2020	20	0	5	9	1	0	0	32
2021	24	0	15	5	1	0	0	42
2022	31	0	12	14	0	0	1	52
2023	26	0	22	13	1	0	0	46
2024	49	0	21	12	5	0	1	77
Total	184	0	108	123	13	0	2	367

Table S1 (Continued). CPT Audit for Immediate Lymphatic Reconstruction and Documented Axillary Lymph Node Dissection

**Panel C. Axillary surgery classification among ILR cases**		
**Axillary surgery classification**	**N**	**% of ILR cases**
Documented ALND	265	72.2%
Sentinel/limited axillary surgery without documented ALND	37	10.1%
Other axillary surgery without documented ALND	43	11.7%
No documented axillary surgery	22	6.0%

**Panel D. CPT composition of documented ALND**			
**CPT code**	**Description**	**ILR cases, n (% of ALND-positive ILR)**	**Overall cohort, n (% of ALND-positive cohort)**
38745	Complete axillary lymphadenectomy	31 (11.7%)	2,027 (16.3%)
19302	Partial mastectomy with axillary lymphadenectomy	5 (1.9%)	423 (3.4%)
19307	Modified radical mastectomy with axillary lymphadenectomy	233 (87.9%)	10,081 (81.3%)
Any documented ALND code	—	265 (100.0%)	12,401 (100.0%)

Note: Documented ALND was defined by the presence of CPT code 38745, 19302, or 19307 in any procedure field. An encounter could contain more than one ALND code; therefore, code-specific counts may exceed the number of unique ALND-positive encounters. CPT code 19307 incorporates modified radical mastectomy with axillary lymphadenectomy.

Table S1 (Continued). CPT Audit for Immediate Lymphatic Reconstruction and Documented Axillary Lymph Node Dissection

**Panel E. Most common CPT combinations among ILR cases**				
**CPT combination**	**N**	**Axillary surgery classification**	**Breast extirpative surgery**	**Recon.**
15777, 19307, 19357, 38900, 38999	9	Documented ALND	Yes	Yes
15777, 19307, 19357, 38999	9	Documented ALND	Yes	Yes
15777, 19307, 19357, 35206, 38308, 38790, 38900, 69990	7	Documented ALND	Yes	Yes
15777, 19303, 19307, 19357, 38999	7	Documented ALND	Yes	Yes
15777, 19303, 19357, 38525, 38900, 38999	7	Sentinel/limited, no documented ALND	Yes	Yes
19307, 19357, 38308, 38790, 38792, 38900	6	Documented ALND	Yes	Yes
19307, 19364, 38308, 38790, 38792, 38900	6	Documented ALND	Yes	Yes
15777, 19303, 19357, 38525, 38999	6	Other axillary, no documented ALND	Yes	Yes
15777, 19303, 19307, 19357, 36590, 38900, 38999	5	Documented ALND	Yes	Yes
15777, 19303, 19357, 38999	4	No documented axillary surgery	Yes	Yes

Abbreviations: ALND, axillary lymph node dissection; ILR, immediate lymphatic reconstruction; Recon., reconstruction.

Counts are unique cases unless otherwise specified. CPT-row counts exceed unique-case counts when a code appears more than once within the same operative encounter. The analytic reconstruction cohort included 65,437 cases, of which 367 had at least one prespecified ILR candidate code.

Table S2: Consolidated Sensitivity and Subgroup Analyses of Immediate Lymphatic Reconstruction

**Analysis**	**N**	**Events / ILR n**	**Adjusted estimate (95% CI)**	**P value**
**Panel A. Any 30-day postoperative complication**				
Primary analysis	65,114	7,078	0.98 (0.71–1.35)	.898
Implant-based reconstruction	55,806	5,546	0.97 (0.67–1.41)	.868
Autologous reconstruction	7,983	1,402	1.05 (0.54–2.03)	.891
Any documented axillary surgery	49,376	5,337	1.06 (0.76–1.47)	.749
Documented ALND	12,320	1,296	0.87 (0.58–1.32)	.518
Contemporary period, 2019–2024	30,292	3,670	0.89 (0.61–1.29)	.539
Strict ILR definition excluding CPT 38999	65,114	7,078	0.94 (0.61–1.47)	.794
ILR code set excluding CPT 35201 and CPT 38305	65,114	7,078	0.98 (0.71–1.35)	.898
Propensity-score matched cohort	1,089	131	0.97 (0.66–1.44)	.897
Expanded 15-covariate Firth-penalized model	64,699	7,030	0.85 (0.61–1.17)	.316
Documented-ALND definition including CPT 38740	65,114	7,078	0.98 (0.71–1.35)	.881
**Panel B. Total operative duration**				
Primary interaction model, 2012–2024	65,055	362	+90.37 min (80.10–100.64)	.796[a]
Contemporary period, 2019–2024	30,279	284	+88.10 min (76.55–99.66)	.195[a]
Excluding highest 1% of operative times	64,404	351	+84.23 min (74.66–93.80)	.110[a]
Any documented axillary surgery	49,330	340	+95.84 min (85.56–106.13)	.973[a]
Documented ALND	12,305	261	+98.93 min (86.45–111.41)	.553[a]
Implant-based reconstruction	55,760	295	+98.19 min (88.80–107.57)	.128[a]
Autologous reconstruction	7,972	64	+54.54 min (12.37–96.72)	.060[a]
Strict ILR definition excluding CPT 38999	65,055	193	+84.69 min (70.68–98.71)	.142[a]

Note: Panel A reports adjusted odds ratios for the composite 30-day complication outcome. Full-cohort models were adjusted for age, body mass index, ASA physical status, reconstruction modality, documented ALND, and operative year; covariates constant within restricted cohorts were omitted. Panel B reports adjusted differences in total operative duration associated with ILR. Positive estimates indicate longer total operative duration among ILR recipients. The operative-duration models included ILR status, operative year, and an ILR-by-year interaction, with the same clinical covariates as appropriate.

[a] P value for the ILR-by-year interaction testing whether the adjusted operative-duration difference changed over time.

Abbreviations: ALND, axillary lymph node dissection; ASA, American Society of Anesthesiologists; CI, confidence interval; CPT, Current Procedural Terminology; ILR, immediate lymphatic reconstruction.

Table S3: Outcomes Associated With Immediate Lymphatic Reconstruction Among Patients With Documented Axillary Lymph Node Dissection

Outcome	N	ILR	No ILR	Unadjusted estimate	Adjusted estimate (95% CI)	P value
Any 30-day complication	12,320	27/262 (10.3%)	1,269/12,058 (10.5%)	0.98 (0.65–1.46)	0.87 (0.58–1.32)	.518
Wound complication	12,320	18/262 (6.9%)	661/12,058 (5.5%)	1.27 (0.78–2.07)	1.02 (0.62–1.68)	.926
Any surgical-site infection	12,320	12/262 (4.6%)	576/12,058 (4.8%)	0.96 (0.53–1.72)	0.77 (0.42–1.39)	.382
Return to the operating room	12,320	17/262 (6.5%)	910/12,058 (7.5%)	0.85 (0.52–1.40)	0.85 (0.51–1.40)	.524
Unplanned 30-day readmission	12,320	12/262 (4.6%)	576/12,058 (4.8%)	0.96 (0.53–1.72)	0.98 (0.54–1.78)	.958
Operative time, min	12,305	328 [255, 435]	220 [166, 290]	+108.0	+98.93 (+86.45 to +111.41)	$< .001$
Length of stay, days	12,316	1 [1, 2]	1 [1, 2]	0.0	−0.07 (−0.55 to +0.42)	.781
Superficial incisional SSI	12,320	6/262 (2.3%)	284/12,058 (2.4%)	0.97 (0.43–2.20)	0.71 (0.31–1.63)	.425
Deep incisional SSI	12,320	5/262 (1.9%)	116/12,058 (1.0%)	2.00 (0.81–4.94)	2.09 (0.82–5.28)	.121
Organ/space SSI	12,320	1/262 (0.4%)	184/12,058 (1.5%)	0.25 (0.03–1.77)	0.20 (0.03–1.47)	.115
Wound dehiscence	12,320	6/262 (2.3%)	116/12,058 (1.0%)	2.41 (1.05–5.53)	2.11 (0.90–4.94)	.085

Note: The analysis was restricted to encounters with documented ALND. Models were adjusted for age, body mass index, ASA physical status, reconstruction modality, and operative year. Documented ALND was omitted because it was constant within the restricted cohort. Odds ratios are reported for binary outcomes; adjusted mean differences are reported for operative time and length of stay.

Abbreviations: ALND, axillary lymph node dissection; ASA, American Society of Anesthesiologists; CI, confidence interval; ILR, immediate lymphatic reconstruction; SSI, surgical-site infection.

Table S4: Exploratory Individual 30-Day Outcomes Associated With Immediate Lymphatic Reconstruction

Outcome	N	Events	ILR	No ILR	Adjusted OR (95% CI)	P value	BH q value
Bleeding	65,114	1,290	15/363 (4.13%)	1,275/64,751 (1.97%)	1.56 (0.90–2.71)	.113	.332
Transfusion	65,114	27	0/363 (0.00%)	27/64,751 (0.04%)	Not estimable	—	—
Venous thromboembolism composite	65,114	330	3/363 (0.83%)	327/64,751 (0.51%)	1.51 (0.48–4.80)	.484	.605
Deep vein thrombosis/thrombophlebitis	65,114	202	3/363 (0.83%)	199/64,751 (0.31%)	2.19 (0.68–7.03)	.188	.332
Pulmonary embolism	65,114	166	1/363 (0.28%)	165/64,751 (0.25%)	1.14 (0.16–8.30)	.897	.968
Pneumonia	65,114	75	0/363 (0.00%)	75/64,751 (0.12%)	Not estimable	—	—
Urinary tract infection	65,114	155	0/363 (0.00%)	155/64,751 (0.24%)	Not estimable	—	—
Superficial incisional surgical-site infection	65,114	1,603	8/363 (2.20%)	1,595/64,751 (2.46%)	0.68 (0.33–1.37)	.279	.399
Deep incisional surgical-site infection	65,114	558	8/363 (2.20%)	550/64,751 (0.85%)	2.43 (1.18–4.98)	.016	.158
Organ/space surgical-site infection	65,114	1,078	4/363 (1.10%)	1,074/64,751 (1.66%)	0.52 (0.19–1.41)	.199	.332
Wound dehiscence	65,114	606	8/363 (2.20%)	598/64,751 (0.92%)	2.00 (0.97–4.11)	.059	.293
Severe complication composite	65,114	1,274	5/363 (1.38%)	1,269/64,751 (1.96%)	0.56 (0.23–1.35)	.196	.332
Systemic sepsis	65,114	299	2/363 (0.55%)	297/64,751 (0.46%)	1.03 (0.25–4.20)	.968	.968
Septic shock	65,114	20	0/363 (0.00%)	20/64,751 (0.03%)	Not estimable	—	—
Unplanned reintubation	65,114	20	0/363 (0.00%)	20/64,751 (0.03%)	Not estimable	—	—

Note: Models were adjusted for age, body mass index, ASA physical status, reconstruction modality, documented ALND, and operative year. The exploratory panel included 15 outcomes. The Bonferroni-adjusted significance threshold was $P < 0.0033$. No outcome met the Bonferroni threshold. Outcomes that could not be estimated had no events among ILR recipients.

Abbreviations: ALND, axillary lymph node dissection; ASA, American Society of Anesthesiologists; BH, Benjamini–Hochberg; CI, confidence interval; ILR, immediate lymphatic reconstruction; SSI, surgical-site infection; VTE, venous thromboembolism.
